# Application of the research domain criteria in early-phase clinical development of transdiagnostic neurotherapeutics: A multidisciplinary perspective

**DOI:** 10.1016/j.nsa.2026.106996

**Published:** 2026-03-07

**Authors:** Igor Magaraggia, Franco Di Cesare, Jordi Alonso, Chris J. Edgar, Pim Heckman, Jenicka Engler, Silvia Zaragoza Domingo, Luca Pani, Rudy Schreiber

**Affiliations:** a Igor Magaraggia Medical Writing and Consulting, Verbania, Italy; b Department of Neuropsychology and Psychopharmacology, Maastricht University, Maastricht, the Netherlands; c Leoben Research Aurora, San Vincenzo Valle Roveto, Italy; d Health Services Research Group, IMIM-Hospital del Mar Research Institute, Barcelona, Spain; e Department of Medicine and Life Sciences, Pompeu Fabra University (UPF), Barcelona, Spain; f CIBER Epidemiology and Public Health (CIBERESP/ISCIII), Madrid, Spain; g PCOA Associates Ltd, London, UK; h Centre for Integrative Neuroscience, Maastricht University, Maastricht, the Netherlands; i Cronos Clinical Consulting Services, Inc., An IQVIA Business, Boston, MA, United States; j Neuropsychological Research Organization S.L. (Neuropsynchro), Barcelona, Spain; k Department of Biomedical, Metabolic and Neural Sciences, University of Modena and Reggio Emilia, Modena, Italy; l Department of Psychiatry and Behavioral Sciences, University of Miami, Miami, FL, United States

**Keywords:** Research domain criteria (RDoC), Precision psychiatry, Transdiagnostic neurotherapeutics, Clinical development, Clinical trials

## Abstract

Transdiagnostic, dimensional frameworks such as the Research Domain Criteria (RDoC) are increasingly regarded as promising vehicles for precision neuropsychiatric drug development, yet no treatment has been approved that was explicitly developed according to such principles. This work, conducted under the aegis of the European College of Neuropsychopharmacology Thematic Working Group on Clinical Outcomes in Early-Phase Clinical Trials, synthesises seven structured multidisciplinary expert meetings supported by a narrative literature review to delineate opportunities and barriers for implementing RDoC in early-phase clinical development. We identify four key operational domains that condition the success of RDoC-aligned programmes: (1) terminology clarity and working definitions for RDoC-aligned trials and target constructs; (2) construct-enriched population selection methodologies; (3) selection, development or modification of construct-aligned clinical outcome assessments that are fit-for-purpose in transdiagnostic research settings; and (4) navigation of regulatory frameworks that remain anchored in categorical diagnoses. Through selected illustrative cases—most notably the aticaprant development program targeting anhedonia in mood and anxiety disorders—we demonstrate how early phase RDoC-aligned trial designs can be compromised at the pivotal stage by the absence of validated endpoints and regulatory constraints on labelling. On this basis, we propose pragmatic recommendations, including consensus-based definitions, registry tagging of RDoC-aligned trials, data-driven biomarker-based transdiagnostic enrichment strategies (i.e., biotyping), and early, iterative engagement with regulators and health technology assessment agencies. Systematic attention to these domains is required for enabling the development of neurobiologically RDoC informed treatments to be delivered to the right patients at the right time.

## Highlights

• Terminological standards for RDoC trials are needed for transparency, harmonization, and synthesis.
• Diagnosis-anchored labels and endpoint rules limit RDoC translation in clinical development.
• Biomarker-based subgrouping enables construct-valid enrichment in RDoC-aligned trials.
• Fit-for-purpose, construct-aligned COAs bridge mechanistic signals and clinical benefit.
• Initial diagnostic anchoring and regulatory engagement enable acceptance of RDoC methods.

## Graphical abstract

**Figure:**
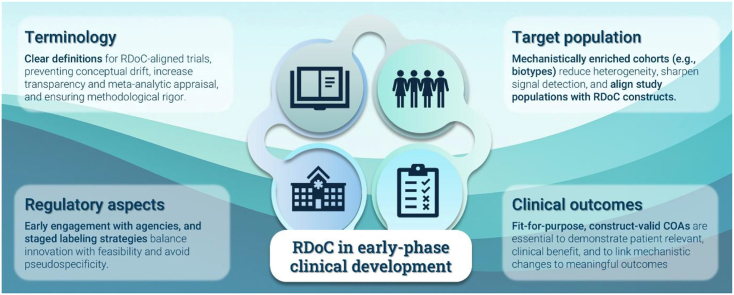
Suggested key operational areas for the application of the Research Domain Criteria (RDoC) in early-phase clinical development of transdiagnostic neurotherapeutics.

## 1 Introduction

Despite decades of progress in neuroscience, the vast majority of investigational neurotherapeutics continue to fail in psychiatric clinical development. While indispensable for clinical care and health system organisation, traditional diagnostic frameworks, such as the Diagnostic and Statistical Manual for Psychiatric Disorders (DSM^1^) and the International Classification of Diseases (ICD), have been widely criticised for fragmenting overlapping pathophysiological processes into discrete categories (Cuthbert and Insel, 2013; Potter and Cuthbert, 2021; Hyman, 2021). These categorical boundaries often obscure shared neurobiological mechanisms and overlook the dimensional features of psychopathology, producing heterogeneous trial populations and attenuated treatment signals (Potter and Cuthbert, 2021; Schreiber and Heckman, 2024). Consequently, promising interventions are frequently tested in unsuitable cohorts, and variability contributes to persistently high failure rates in psychiatric drug development compared to other therapeutic areas (Zhu, 2021; Butlen-Ducuing et al., 2016).

In recent years, several attempts have been made to overcome the limitations of current diagnostic frameworks. Above all, the Research Domain Criteria (RDoC^2^) seeks to leverage innovations in neuroscience methodologies to restructure psychopathology into dimensional, neurobiologically-anchored constructs, while considering transdiagnostic phenomena that cut across conventional diagnostic boundaries (Cuthbert and Insel, 2013; Fusar-Poli et al., 2019; Insel et al., 2010; Morris et al., 2022). This approach resonates with the broader ambitions of precision psychiatry by enabling the stratification of patients into subgroups defined by construct-relevant features rather than categorical diagnoses (DSM/ICD). By providing a robust platform for precision psychiatry research, RDoC may ultimately facilitate the development of more effective neurotherapeutics delivered to the right patient at the right time (Williams et al., 2024; Insel, 2014).

Since its development, a vast number of studies have successfully investigated psychopathological conditions using the RDoC framework (Pacheco et al., 2022). However, the application of the RDoC framework in clinical development remains in its infancy, with currently no approved treatment developed explicitly according to its principles (Potter and Cuthbert, 2021; Potter et al., 2024). Its inherent complexity and paradigm-shifting nature make it difficult for RDoC to be integrated within a clinical research landscape fundamentally structured around categorical diagnoses and lacking formalised implementation guidelines for precision psychiatry approaches. Therefore, systematically identifying the obstacles that its integration is facing is essential to stimulate the implementation of RDoC in routine clinical development.

To examine these challenges, we conducted a narrative review informed by 7 structured online expert meetings between March and September 2025, convened under the European College of Neuropsychopharmacology Thematic Working Group on Clinical Outcomes in Early-Phase Clinical Trials (Clinical Outcomes in Early-Phase Clinical Trials - ECNP). These meetings brought together a multidisciplinary panel of psychiatrists, neuropsychologists, psychopharmacologists, regulatory agents, public health experts and clinical outcome assessment (COA) scientists, ensuring that diverse perspectives were captured. Rather than offering prescriptive solutions, our goal was to stimulate methodological reflection and chart a collaborative agenda for future work needed for the implementation of RDoC in clinical development.

Discussions were synthesised into consensus threads, which highlighted 4 key operational areas presenting barriers to the implementation of RDoC in clinical development: (i) terminological clarity and definitions for RDoC-aligned trials, (ii) selection of construct-enriched target populations, (iii) clinical outcome assessments, and (iv) current regulatory frameworks. Given the small and heterogeneously reported pool of interventional studies extracted following an initial search using the search term “Research Domain Criteria”, a formal systematic review was considered unfeasible. Instead, an expert-informed narrative review, supported by iterative searching in PubMed and ClinicalTrials.gov, was conducted to identify literature relevant to RDoC constructs, biomarker families, and candidate therapeutics. Search terms included combinations of ‘RDoC/Research Domain Criteria’, transdiagnostic/precision psychiatry, relevant domains/constructs (e.g., anhedonia), clinical trial enrichment strategies (e.g., ‘biotyping’, biomarker-enriched sampling) and illustrative interventions (e.g., aticaprant, navacaprant, brexanolone). Illustrative case studies were selected based on prior knowledge and validated against primary publications and trial registries to provide concrete examples of key implementation barriers. and are illustrated throughout the manuscript.

Of note, our focus was on early-phase clinical development due to the absence of late-phase clinical trials explicitly informed by RDoC, and the exploratory nature of early-phase trials that provides flexibility to test novel methodologies and study designs. Yet, our reflections inevitably project onto late-phase trials as well, since strategic planning of early-phase clinical development is ideally informed by future clinical application of novel treatments. Before discussing the key operational areas identified during our meetings, the next section will first provide to reader with an overview of the current state of the art around RDoC's implementation in early-phase clinical development of novel neurotherapeutics.

## 2 RDoC in early-phase clinical trials

Early-phase clinical trials are pivotal in neurotherapeutic development, as they provide initial evidence of treatment safety and efficacy in humans. As such, careful planning and robust methodology in early-phase studies are essential to minimise downstream risks and increase the likelihood of successful translation in psychiatric drug development. In fact, weaknesses at this stage have repeatedly predicted costly failures in later stages of development (Butlen-Ducuing et al., 2016).

Against this backdrop, the RDoC framework is particularly well-suited to de-risk early-phase development programmes. Conceived as a translational research paradigm, RDoC's dimensional logic maps naturally onto proof-of-mechanism and proof-of-concept studies, where the goal is to validate biological targets and link neural systems to functional outcomes (Schreiber and Heckman, 2024). It is widely acknowledged that establishing a clear hypothesis about how a treatment is expected to modulate a pathophysiological mechanism to provide clinical benefit can reduce the risk of selecting inappropriate patient populations or dosing regimens, thereby avoiding exposing patients to ineffective interventions. Similarly, the lack of a clear hypothesis could lead to the selection of outcomes that are not fit-for-purpose (FFP) which, in turn, may cause reduced detection of efficacy signals. RDoC's multimodal framework—spanning molecular, cellular, circuit-level, physiology, behavioural, and self-report levels of analysis—yields convergent mechanistic readouts that can inform study design, including adaptive dosing and more accurate go/no-go decisions in pivotal development phases (Potter and Cuthbert, 2021; Magaraggia et al., 2025). As a result, implementing RDoC in early-phase clinical development has the potential to reduce human burden, shorten timelines and reduce costs associated with later-stage drug development failures.

**Fig. 1:**
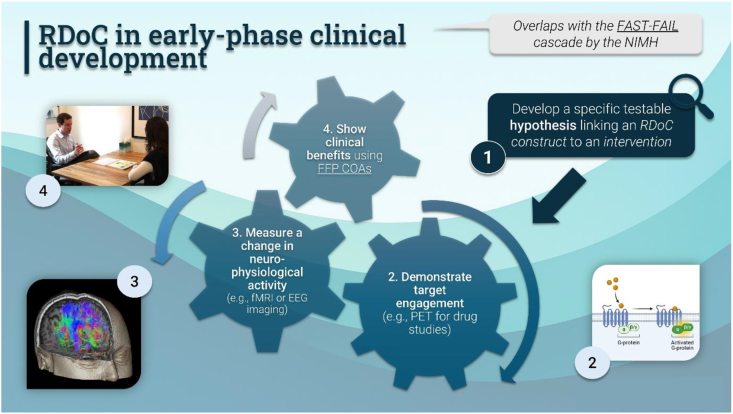
Overview of how the application of the RDoC framework to early-phase clinical development aligns with the NIMH FAST-FAIL cascade, linking mechanistic hypotheses to multi-level evidence of treatment effects. Early development proceeds through: (1) formulating an RDoC-based hypothesis, (2) demonstrating target engagement (e.g., PET), (3) showing neurophysiological change (e.g., fMRI/EEG), and (4) quantifying clinical benefit via early-phase, FFP COAs—thereby de-risking translation from mechanism to patient-relevant outcomes. Abbreviations: Research Domain Criteria (RDoC); National Institute of Mental Health (NIMH); Positron Emission Tomography (PET); functional Magnetic Resonance Imaging (fMRI); Electroencephalogram (EEG); Fit-For-Purpose (FFP); Clinical Outcome Assessments (COAs).

Recent initiatives provide valuable insights into the practical implications of RDoC in early-phase clinical development. Most notably, the FAST-FAIL programme launched by U.S. National Institute of Mental Health in 2012 was explicitly developed according to RDoC's dimensional and transdiagnostic principles (Grabb et al., 2016, 2020). It consisted of series of small, mechanistically focused early-phase studies aimed at rapidly confirming (or refuting) biological target engagement, neural-circuit modulation, and short-term clinical signals before larger investments are made (Grabb et al., 2016, 2020; Szabo et al., 2015). Typical FAST-FAIL trials incorporated various biomarkers, from molecular to neuroimaging and behaviour, conceptualized as a sequential cascade, as illustrated in Fig. 1, where each stage links mechanistic hypotheses to multi-level evidence of treatment effects. By front-loading mechanistic validation, the initiative aimed to accelerate translation of novel mechanisms into later-phase trials while conserving resources.

Among the FAST-FAIL programmes, the early development programme of the κ-opioid receptor (KOR) antagonist aticaprant for the treatment of anhedonia (i.e., the inability to experience pleasure or enjoyment from activities that would normally be pleasurable) exemplifies how RDoC principles may support decision-making in early-phase psychiatric drug development (Serretti, 2023). After an initial positron emission tomography (PET) study demonstrating KOR near-saturation at clinically tolerable doses of the drug (Naganawa et al., 2016), the FAST-MAS 8-week, double-blind Proof-of-Mechanism Phase 1b trial investigated the effects of aticaprant on anhedonia conceptualized within RDoC's Positive Valence system—more specifically the “Reward Responsiveness” subconstruct (Krystal et al., 2020; Pizzagalli et al., 2020). Consistent with anhedonia's transdiagnostic nature, the FAST-MAS study recruited patients with elevated symptoms across various DSM-recognised mood and anxiety disorders, such as Major Depressive Disorder (MDD), Generalized Anxiety Disorder, and Post-Traumatic Stress Disorder (Krystal et al., 2020). To test the hypothesis of anhedonic symptom reduction through KOR inhibition in the ventral striatum, the study employed multi-modal assessments methods comprising of neuroimaging, behaviour, and self-reports measures.

The results of the FAST-MAS trial were positive. Compared to placebo, aticaprant was found to increase ventral-striatal activation during the Monetary Incentive Delay task (primary endpoint) (Knutson et al., 2000), improve anhedonic symptoms on the Snaith-Hamilton Pleasure Scale (SHAPS; secondary endpoint) (Snaith et al., 1995), and showed a signal on the Probabilistic Reward Task (Pizzagalli et al., 2008) mean response-bias metric (i.e., a behavioural proxy measure for reward deficits) (Krystal et al., 2020; Pizzagalli et al., 2020). These results justified further investment in aticaprant's development as a treatment for anhedonia in a subsequent Phase 2a Proof-of-Concept trial in patients with a primary MDD diagnosis and inadequate response to first-line SSRI treatment. The results of the latter study confirmed the preliminary efficacy data from the FAST-MAS trial (Schmidt et al., 2024) and, considering the high failure rate of novel psychiatric interventions in Phase 2 trials, illustrate how RDoC-guided FAST-FAIL designs and endpoints can de-risk early-phase clinical development (Potter et al., 2024).

The aticaprant program also hints to specific challenges of operationalizing RDoC at pivotal stages of the clinical development pipeline. In fact, despite its successful early-phase development, aticaprant's late-phase development as adjunctive treatment for MDD encountered a significant drawback. Due to a preliminary lack of efficacy, the VENTURA Phase 3 trial was halted in March 2025 (Johnson, 2025). Considering various factors that may have led to this negative outcome (Mullard, 2025), our group regards aticaprant's development programme as an illustrative case highlighting both the promises and the systemic hurdles that must be addressed for the effective translation of the RDoC framework into clinical development. In a similar vein, although not explicitly following RDoC principles, we identified additional case examples illustrating systemic challenges to RDoC implementation in clinical development. All illustrative cases are enumerated and critically evaluated in Table 1, with further in-depth analyses provided in Supplementary Table S1.

**Table 1:** Illustrative clinical development case studies highlighting opportunities and pitfalls for RDoC-aligned programmes.

Intervention	RDoC construct(s) / Mechanistic target	Early-phase RDoC-aligned readout	Pivotal endpoint / regulatory interface	Core lesson for RDoC implementation	Key primary sources
Aticaprant (JNJ-67953964) κ-opioid receptor antagonist	Positive Valence Systems: Reward valuation/learning; anhedonia	Fast-Fail proof-of-mechanism: ↑ ventral striatal reward-anticipation fMRI + SHAPS/behavioral signal	Phase 3 (VENTURA) anchored to DSM-MDD; MADRS primary; programme discontinued for insufficient efficacy	Early biomarker/construct alignment can be lost when Phase 3 reverts to standard DSM endpoints and broader populations	(Krystal et al., 2020; Schmidt et al., 2024; Johnson, 2025)
Navacaprant (NMRA-140) κ-opioid receptor antagonist	Positive Valence Systems: Anhedonia/reward processing	Phase 2 targeted anhedonia signal; SHAPS included as endpoint	Phase 3 KOASTAL-1: MADRS primary + SHAPS key secondary; no significant benefit in ITT; ongoing programme reassessment	Construct-relevant endpoints may still fail to translate; placebo response/subgroup effects highlight need for enrichment and replication	(Neumora; Mathew et al., 2025)
Brexanolone (IV allopregnanolone) GABA-A positive allosteric modulator	Arousal/Regulatory Systems: Stress/hormone-linked affect regulation	Large rapid HAM-D improvement after 60–72 h infusion in severe postpartum depression trials	FDA approval (2019) yet high-burden delivery; FDA withdrawal of NDA in 2025 after product no longer marketed	Even with strong mechanistic rationale and efficacy, deployability and system constraints can block scalable impact	(Kanes et al., 2017; Federal Register: Sage Therapeutics and Inc)
ALTO-100 (NSI-189 phosphate) Neurogenic small molecule	Cognitive Systems: Learning/memory; cognitive impairment in depression	Early studies suggested cognitive/functional signal despite mixed MADRS effects	Later biomarker-stratified trials pursued; Phase 2b did not meet primary endpoint in biomarker-defined subgroup	Illustrates biomarker-guided enrichment ambition + fragility: predictive signatures need replication and prespecification	(Alto Neuroscience and Inc; Papakostas et al., 2019; Fava et al., 2015)
rTMS + ERP in OCD Task-fMRI predictors	Cognitive Systems: Cognitive control (planning/inhibition)	Task-based fMRI activation/connectivity associated with response to specific rTMS targets (proof-of-concept)	Not yet regulatory-facing; demonstrates path toward mechanistically interpretable predictors for treatment selection	Predictive neural markers may be target-specific; mechanistic interpretation and feasibility need confirmatory testing	(Postma et al., 2025; Fitzsimmons et al., 2025)

Abbreviations: Research Domain Criteria (RDoC); functional magnetic resonance imaging (fMRI); Snaith–Hamilton Pleasure Scale (SHAPS); Diagnostic and Statistical Manual of Mental Disorders (DSM); major depressive disorder (MDD); Montgomery–Åsberg Depression Rating Scale (MADRS); intention-to-treat (ITT); gamma-aminobutyric acid (GABA); U.S. Food and Drug Administration (FDA); New Drug Application (NDA); repetitive transcranial magnetic stimulation (rTMS); Hamilton Depression Rating Scale (HAMD/HAM-D); exposure and response prevention (ERP); obsessive-compulsive disorder (OCD).

## 3 Terminology clarity and definitions for RDoC-aligned trials

In drug development, alignment on terminology and definitions is of utmost importance to enhance effective communication and understanding among stakeholders. During our initial discussions, we recognised that terminological ambiguity around the definition of “RDoC-aligned trials” represented a key obstacle for effectively discussing the challenges for the implementation of RDoC in clinical development, in particular when screening available literature for clinical trials following the RDoC's logic.

According to the RDoC official website, any experiment probing dimensional constructs “across the health-to-illness continuum” *could* qualify as an RDoC study (or trial) yet admits there are “no rigid criteria” for determining what constitutes such a study in practice (RDoC Frequently Asked Questions (FAQ)). While conceptual openness encourages methodological innovation, it is also vulnerable to operational risks. For example, conventional trials could be labelled as RDoC-aligned without following fundamental principles underlying its approach (e.g., clearly identifying the target constructs), thereby diluting the specificity of this framework. Similarly, enthusiasm for “multiple units of analysis” can foster unwieldy protocols in which exploratory endpoints proliferate without an evidentiary hierarchy and are underpowered from the statistical standpoint to find any significant results. The risk of inconsistent application of the RDoC framework may ultimately undermine its perceived rigour and credibility within the scientific and regulatory communities.

Experience from the wider transdiagnostic literature underscores the risks of lacking clear terminology. A systematic review of studies self-described as “transdiagnostic” catalogued several issues across programs derived from this lack of clear definitions, such as inconsistent diagnostic anchors, vague outcome definitions, and heterogeneous conceptual rationales. To correct such terminological drift, the authors proposed the TRANSD criteria for transdiagnostic studies which have now become a standard in the field (Fusar-Poli et al., 2019; Fusar-Poli, 2019). Borrowing that logic, we argue that RDoC-aligned trials should use an equally explicit working definition—one that stipulates fundamental RDoC concepts such as construct designation, enrichment strategy, endpoint selection, and multi-level assessment (i.e., units of analysis). In short, codifying what “classifies” as an RDoC trial is no mere semantic exercise; it is a prerequisite to generate interpretable data and test the framework's promise to de-risk neuropsychiatric drug development.

### 3.1 Towards a working definition for RDoC-aligned clinical trials

Considering the demonstrated gains seen after the introduction of CONSORT and SPIRIT extensions for specialised trial designs (Hopewell et al., 2025; Altman, 1996; Chan et al., 2025), we reached consensus on the necessity of establishing a clear definition for RDoC-aligned early-phase trials and propose the following working definition for RDoC-aligned trials. According to the RDoC principles, an RDoC-aligned early-phase trial might be one that:

(i) Designates an RDoC matrix sub-construct (e.g., reward responsiveness) as its primary target.
(ii) Enriches or stratifies its sample on mechanistic criteria linked to that construct rather than on categorical diagnosis alone.
(iii) Embeds a multi-level measurement model (e.g., spanning at minimum one neurobiological and one behavioural or self-report unit of analysis, all anchored to the same construct.)
(iv) Prespecifies decision rules that relate those mechanistic read-outs to clinically meaningful outcomes.

This rubric is offered not as a definitive standard but as a concrete illustration of the elements that will likely be required when the field converges on a formal definition of RDoC-aligned trials. Once these criteria are defined, clinical trial public registries (e.g.*,* ClinicalTrials.gov, EU-CTR) may consider introducing an “RDoC” tag or keyword field to facilitate empirical testing and meta-analytic appraisal of such studies. A registry tag would make RDoC trials readily discoverable by scientists and sponsors would enable sharing the most recent experiences allowing replication, as well as systematic evidence aggregation. It would also promote transparency by requiring investigators to declare construct alignment and multi-level endpoints at the point of registration (Kanes et al., 2017; Federal Register Sage Therapeutics and Inc; Alto Neuroscience and Inc).

### 3.2 Construct definition

In drug development, terminological clarity is essential also for defining and validating clinically relevant transdiagnostic constructs. Once again, the case of anhedonia illustrates this problem vividly. In fact, anhedonia is a transdiagnostic phenomenon that is frequently conflated with related but distinct constructs such as apathy, amotivation, and avolition (Davidson et al., 2025; Husain and Roiser, 2018; Strauss and Cohen, 2017). While these terms may overlap in their clinical presentation, they represent different phenomena and thus different positions within the RDoC matrix. Anhedonia, for example, maps primarily onto the Positive Valence domain, specifically the Reward Responsiveness and Reward Learning subconstructs (Krystal et al., 2020). Apathy, in contrast, is broader and cuts across motivational and executive processes (Miller et al., 2021). The variety in the use of similar terminology in clinical practice, has a shortcoming when using more accurate mechanistic frameworks. Therefore, without terminological precision, enrichment strategies for sample selection may inadvertently select heterogeneous patient populations and mislead the use of the right outcome measures failing to capture the intended construct. This undermines face and construct validity, complicates regulatory dialogue, and reduces the translational value of trial results.

Consensus-building activities among key stakeholders may play a crucial role in establishing clear and operational definitions for RDoC constructs. For example, the International Society of CNS Trials and Methodology (ISCTM) Apathy Working Group used a modified Delphi methodology to establish diagnostic criteria for Apathy in neurocognitive disorders, involving experts from academia, industry, and regulatory agencies (Miller et al., 2021). While acknowledging the inherent limitations of consensus-based methodologies stemming from their reliance on expert opinion, potential for bias, and the sociological dynamics of group interaction (Hsu and Sandford, 2007; Murphy et al., 1998), this initiative underscores both the feasibility and value of such methods to characterize neurobehavioural constructs. Ultimately, the goal is to ensure that definitions of constructs are transparent, reproducible, clinically relevant, and endorsed across stakeholders before their implementation in clinical programs.

## 4 Selecting construct-enriched target populations

In psychiatry, inadequate target population selection remains one of the most consistent predictors of New Drug Application failures at regulatory agencies (Butlen-Ducuing et al., 2016), which underscores the need for rigorous selection strategies in clinical trials. Due to its categorical nature, DSM/ICD-based recruitment in clinical trials often leads to heterogeneous patient populations with diverse underlying pathophysiology, who thus respond differently to treatment. With its dimensional and transdiagnostic logic, the RDoC framework tries to overcome these issues by focussing on biologically anchored neurobehavioural constructs and their dysfunctions. According to this principle, RDoC-aligned clinical trials ideally should select and recruit patients based on measurable and clinically relevant construct dysfunctions. However, official guidance on how to identify, validate, and operationalize constructs dysfunctions as inclusion criteria for RDoC-aligned trials is still missing.

### 4.1 Biotyping: promises and current gaps

Various innovative enrichment strategies have proven to be suitable candidates to identify, validate, and operationalize RDoC constructs in clinical trials. These enrichment strategies are based on the data-driven identification of patient subgroups through convergent multi-level biomarkers (e.g., genomic, molecular, neuroimaging) (Williams et al., 2024; Zhang et al., 2023). Commonly referred to as ‘biotyping’, these approaches have already shown translational potential in major consortia, such as EMBARC, PReDICT and B-SNIP.

The EMBARC study stratified individuals with major depressive disorder based on dysregulation in the “loss” and “sustained threat” subconstructs of RDoC's Negative Valence Systems domain, using cortisol dynamics and functional connectivity as physiological and circuit-level read-outs (Trivedi et al., 2018). Similarly, the PReDICT trial demonstrated that heightened amygdala reactivity—a circuit-level indicator of the acute threat construct—could differentiate cognitive-behavioural therapy responders from SSRI responders across anxiety–depression spectra (Dunlop et al., 2017; Dunlop, 2015). In a transdiagnostic pool of psychosis-related disorders (e.g. Bipolar Disorder, Schizophrenia, Schizoaffective Disorder), the B-SNIP programme generated three distinct biotypes by clustering electroencephalography (EEG) sensory-gating deficits, cognitive-control event-related potentials, and structural Magnetic Resonance Imaging (MRI) measures (Clementz et al., 2016, 2022). Interestingly, preliminary data suggested a uniquely favourable response to clozapine of one biotype compared to the others, a finding now under prospective evaluation in a multicentre trial (NCT04580134). Taken together, these studies demonstrate how biotypes can operationalize the multi-levelled taxonomy of the RDoC matrix into concrete population definitions, moving beyond symptom checklists toward mechanistic homogeneity (Clementz et al., 2024).

Cognition-centred projects provide further support for this strategy. The BIG-Depression study stratified patients according to intrinsic connectivity networks linked to executive function, identifying selective benefits of alpha-2A adrenoceptor agonism (Williams et al., 2025). Likewise, the TULSA-1000 cohort quantified multiple RDoC domains across 1000 outpatients with mood, anxiety, substance-use, and eating disorders, generating a multidimensional fingerprint that supports construct-based recruitment for proof-of-concept trials (Burrows et al., 2021; Victor et al., 2018). In parallel, the RDoC Anxiety-Depression project has mapped acute threat, loss, and sustained threat constructs onto daily-function outcomes using ecological momentary assessment and multimodal imaging, offering scalable algorithms for identifying transdiagnostic intervention targets (Williams et al., 2016). Collectively, these initiatives exemplify the growing research commitment to identifying and/or validating constructs through biotyping enrichment strategies, with results to date frequently demonstrating promising success.

Despite encouraging proof-of-concept data, several obstacles impede the routine adoption of biotyping in clinical trials. First, most biotypes have been derived from single-centre datasets and larger, standardized, and harmonized multi-site studies are needed to prove and validate construct stability across different clinical populations, environmental conditions and contexts of use. Second, the field lacks consensus standards for integrating multimodal metrics—ranging from EEG power spectra to digital phenotyping—into common quantitative units, complicating pooled analyses. Third, regulatory agencies increasingly expect explicit evidentiary frameworks that link biotype assignment to adaptive study design decision rules (European Medicines Agency (EMA), 2025; U.S. Food and Drug Administration, 2019). Fourth, patient-centred drug-development principles demand evidence that biotype labels correspond to clinically relevant symptoms that are meaningful to the patient's ability to feel, function, or survive. Finally, biomarkers used for stratification must satisfy analytical and clinical validity criteria acceptable to regulators, underscoring the importance of early scientific-advice interactions (see later sections of the manuscript).

### 4.2 Transdiagnostic recruitment and the risk of pseudospecificity

Transdiagnostic recruitment is central to the RDoC's logic: instead of restricting enrolment to a single primary DSM/ICD disorder, patients are recruited across diagnostic boundaries according to measurable dysfunctions within a given construct. This approach better captures shared neurobiological substrates and aligns with how symptoms manifest in real-world populations, where construct comorbidity across diagnoses is the rule rather than the exception. In fact, there is a growing interest in exploring the effects of novel interventions in transdiagnostic pools of patients. Besides the previously mentioned FAST-MAS trial (Krystal et al., 2020), other early-phase trials have utilised this recruitment strategy in their study design. For example, an open-label Phase 1b trial with psilocybin (NCT06442423) is currently enrolling adults with at least one psychiatric condition causing with functional impairments due to mood, anxiety, trauma, or addiction disorders in a diagnosis-agnostic fashion, excluding primary psychotic disorders.

Although transdiagnostic recruitment holds promise for simultaneously testing the efficacy of treatments targeting shared constructs across disorders, its appeal is tempered by the risk of pseudospecificity. In psychiatric drug development, pseudospecificity refers to artificially narrow claims of efficacy for a symptom domain when effects likely reflect broader improvement in general psychopathology rather than a true, independent target (Goldberg, 2024). This is a key regulatory concern for transdiagnostic trials, where changes in the targeted construct (such as agitation or anhedonia) may be secondary to global symptom relief (Forum on Neuroscience and Nervous System Disorders et al., 2015). Therefore, simply enrolling ‘agitated’ or ‘anhedonic’ patients across disorders can recapitulate DSM-level heterogeneity in treatment response if the underlying pathology differs.

To minimise the risk of pseudospecificity, transdiagnostic recruitment should, in our view, integrate convergent biomarker evidence across RDoC units of analysis of the given construct—for example, pairing a behavioural task with functional Magnetic Resonance Imagingread-outs. Only when symptom expression aligns with consistent underlying changes at various levels of analysis similarly across different DSM/ICD conditions can a transdiagnostic cohort be said to represent a construct-anchored population. Construct-valid multimodal biomarker enrichment is therefore a prerequisite, not a luxury, to truthfully operationalize RDoC's dimensional and transdiagnostic ethos.

## 5 Transdiagnostic approach for clinical outcome assessments

Clinical outcome assessments (COA) occupy a central role in clinical development to demonstrate the clinical benefits of novel interventions for regulatory approval. For this purpose, regulators require COA tools that are fit-for-purpose (FFP); meaning that they measure what they are meant to measure demonstrating content validity, reliability, responsiveness, and clinical meaningfulness within the intended clinical context of use (referring to the target clinical population, its disease stage, etc.). Thus, without a valid COA strategy, the mechanistic promise of an RDoC-based intervention remains theoretical because it cannot be translated into a clinical utility of benefit.

Considering their importance, standards for the effective selection, development or modification of FFP Clinical Outcome Assessments have been developed and are guided by the FDA's Patient-Focused Drug Development (PFDD) principles (U.S. Food and Drug Administration, 2025). These standards have recently been tailored for clinical trials in psychiatry and neurology using an operationalized methodology (Zaragoza Domingo et al., 2024). A core premise of all standards is the definition of a clearly articulated conceptual foundation, notably the conceptual disease model and related concepts of interest (COI) to be measured (i.e. signs, symptoms, and impacts) based on qualitative research with patients living with specific medical conditions.

When selecting a COA strategy, the conceptual disease model serves as the basis to decide which COIs should be measured in connection with the intervention's mechanism of action and the sponsor's strategy to differentiate from competitors. This step, defined as deciding on “What to Measure”, is critical and usually targets those COIs that are most burdensome to patients, and where there is an unmet medical need, defining a set of outcomes and their hierarchy in the study design (i.e., primary, co-primary, secondary or exploratory endpoints). RDoC introduces a fundamental shift in how a disease model is defined, which challenges current standards for COA selection, development, or modification. Instead of relying on categorical classifications of diagnoses, RDoC focuses on clinically relevant transdiagnostic constructs (e.g., motivational deficits in mood and anxiety disorders) (Morris et al., 2022; Insel, 2014). When developing a COA strategy, this construct represents the COI to be measured to demonstrate the clinical utility of the investigational treatment. Essentially, the definition and clinical meaningfulness of a COI should be co-developed with patients and/or their representatives according to PFDD guidelines (Clementz et al., 2024; Williams et al., 2025).

Once an operational definition and standardization of the COI is achieved, the next step of the COA selection process focuses on “How to Measure” the COI. FDA regulatory guidance suggests that existing COA measures for which there is already experience in the relevant context of use are generally preferred, particularly when measuring well-established COIs (e.g., pain intensity). In such cases, sponsors should summarize existing information and evidence that supports the rationale for the use of the COA and assess how well the rationale is supported by the available information in the intended context of use. Furthermore, the use of existing items from item pools or instruments following patient-centred methodology (e.g., PROMIS or C-PATH) may support building and validating new measures for clinical practice and research (Cella et al., 2010, U.S. Food and Drug Administration, 2025).

It is worth mentioning that RDoC's online repository provides a list of tools for measuring each of its constructs, including behavioural tasks and self-reports. However, these tools are often experimental in nature and may therefore not be FFP for demonstrating the clinical utility of a novel intervention in pivotal efficacy trials. Moreover, as many of these tools are inherited from DSM-centred research (U.S. National Institute of Mental Health (NIMH), 2016), they may require modification and validation in transdiagnostic settings. Nevertheless, they may be used as a starting point to draw a validation roadmap for specific use in transdiagnostic drug development, or to inform the development of novel instruments. In case of the latter, measurement scientists may consider different types of instruments (e.g., traditional scales migrated to electronic handheld devices, passive data collection through smart digital devices), or even hybrid mixing different approaches depending on the requirements and intended context of use (Elmer et al., 2025; Mengelkoch et al., 2024; Stinson et al., 2022).

### 5.1 Attempts and case examples

Core outcome sets (COS) are consensus-derived minimum bundles of outcomes that studies in a therapeutic area should measure and report, improving cross-trial comparability, reducing outcome-reporting heterogeneity, and strengthening the evidentiary value of clinical development programs. Attempts to develop COS in transdiagnostic psychiatry can be found although these are not frequent. For instance, the emerging Patient Important Outcomes in Psychiatry (PIO-Psych) initiative developed a protocol aiming to identify clinical outcomes important to patients and not tied to one specific mental health condition (Juul et al., 2025). Other examples can be found targeting concepts such as cognition and pain (Williamson et al., 2017).

Another exemplar of the opportunities arising from international collaborations for COA development is the Reward Task Optimisation Consortium (RTOC). This industry-academic partnership is supported by ECNP's Experimental Medicine Network (Experimental Medicine Network - ECNP) and aims to standardise three computerised reward-processing tasks (paired with EEG read-outs) for use as objective, transdiagnostic measures of motivational deficits in major depressive disorder and schizophrenia (Bilderbeck et al., 2020; Malik et al., 2025). Unlike legacy self-report scales, the RTOC battery was designed from the outset to meet regulatory data-integrity requirements and to operate in large, multi-site trials. Although these tasks themselves could not form the basis for regulatory approval without complementary patient-centred outcomes, RTOC provides a valuable proof-of-principle for multi-level task development for dimensional and transdiagnostic constructs.

In conclusion, the selection, development, or modification of COAs for RDoC-aligned trials requires addressing conceptual, methodological, and regulatory challenges concurrently. To provide an overview of these challenges, Table 2 offers an illustrative set of guiding questions for clinical developers who aim to implement the RDoC approach in their development plan. These questions underscore that selecting and validating COAs in RDoC contexts is not only a technical exercise but also a process of ensuring that constructs remain clinically meaningful, valid in transdiagnostic settings, and regulatorily defensible. Embedding such structured reflection into early development is essential to transform mechanistic innovation into demonstrable patient benefit. For additional considerations regarding COA selection and development in RDoC settings, the reader is referred to the Supplementary Table S2.

**Table 2:** Examples of guiding questions for clinical developers when selecting COAs in RDoC-aligned trials.

For biologically defined transdiagnostic entities …	When specifying an endpoint …
What areas of overlap or difference in the patient experience exist between different syndromic groups based on prior clinical diagnostic criteria?	Is there an existing COA that is valid and FFP in the intended context of use?
Is a new conceptual model required? If yes, should this require an in-depth exploration of the transdiagnostic concepts of interest and context of use incorporating patient perspectives?	Is modification or development work required?
Is the patient experience of a concept of interest for measurement sufficiently homogenous across, diagnostics, genders and cultures?	What evidence is there for bridging between early phase and late phase clinical development (i.e., if different, to what extent are endpoints used in early phase for signal detection, predictive of those used in registrational trials to demonstrate treatment benefit)?
	Is the same MCID valid in all subsamples? Should anchors used for the definition of MCID be specific for each subpopulation?
	Is the same cut-off score useful in identifying all subsamples? Equal sensitivity and specificity to screen for the targeted concept?
	Are psychometric properties of the COA adequate for all planned contexts of use/subsamples (construct validity, convergent-divergent, etc).
	Are Rash methods applicable when selecting items in a transdiagnostic context?
	Are anchors used for the definition of MCID specific for each subpopulation?

Abbreviations: clinical outcome assessment (COA), fit-for-purpose (FFP), minimal clinically important difference (MCID).

## 6 Regulatory aspects

Embedding the RDoC framework into clinical development represents a paradigm shift that significantly challenges traditional regulatory frameworks. Above all, the RDoC's construct-centred development of treatments represents a major hurdle for drug labelling, which is still reliant on conventional DSM/ICD diagnoses. At present, both FDA and EMA expect indications to be stated in terms of a *recognised disease or condition* or a *well-defined manifestation of a recognised disease* (FDA 21 CFR 201.57; EMA wording principles). Novel, purely construct-based labels (e.g., “anhedonia across mood and anxiety disorders”) currently lack precedent. Therefore, until construct dysfunctions are officially recognised as independent disease entities by regulators, developers should seek alternative labelling strategies for their RDoC-based therapeutic.

Despite the lack of clear precedents, past approvals provide useful analogies that may inform the development of “regulators-FFP” labelling strategies for RDoC-based therapeutics. In a few cases, drugs have been labelled for symptom-based indications tied to a particular context of use (e.g., aripiprazole's indication for “irritability associated with autistic disorder” in paediatric patients (Owen et al., 2009), or pimavanserin's indication for “hallucinations and delusions associated with Parkinson's disease psychosis” (Cummings et al., 2014)). In these instances, the symptom or domain (irritability, psychosis) is explicitly linked to a specific DSM/ICD condition (Autistic Disorder, Parkinson's Disease) in the labelling. Such precedents show that regulators can countenance non-traditional outcomes, but only with a clear case that the symptom is a well-characterized manifestation of an established disorder and that clinical trials have used validated measures of that symptom to show benefit (Owen et al., 2009; Cummings et al., 2014).

Considering the above, an RDoC-aligned development program may need to pursue a strategy where a transdiagnostic construct is operationalized in terms of a subset of patients within a recognised disorder, at least initially. For example, exploratory early-phase trials might enrich for patients with a high level of anhedonia across major depression and anxiety disorders, but the sponsor could seek approval first in “MDD with prominent anhedonia,” and later expand labelling to other indications with additional registrational studies (e.g., in Generalized Anxiety Disorder patients with elevated anhedonia). This approach anchors the indication in a conventional category while leveraging the transdiagnostic finding (as was partly done in pivotal trials of the aticaprant program (Schmidt et al., 2024);). Within this anchored framework, developers can still prospectively incorporate construct-relevant assessments as secondary endpoints and pre-specified subgroup analyses, thereby generating construct-level evidence without over-relying on exploratory claims. In practice, an anchor-first approval becomes a platform for later expansion: post-approval or follow-on studies can extend use to adjacent populations or to subgroups defined by an RDoC trait as the mechanism–benefit link matures, analogous to stepwise label growth often seen in oncology (Marcus et al., 2019).

As previously noted, another fundamental requirement for regulatory agencies is that biomarkers and clinical outcome measures used in pivotal trials must be qualified as FFP. Recent EMA publications underscore the importance of “grounding [the] validation strategy” in established measures and defining a clear context of use when seeking regulatory qualification of a novel biomarker or endpoint (Drmić et al., 2024). This is particularly relevant for RDoC clinical development settings, considering that regulators may scrutinize whether a construct-based transdiagnostic outcome measure performs consistently across heterogeneous subgroups. If a scale or biomarker functions differently in, say, patients with depression versus those with anxiety (e.g., different placebo response or variance), it could undermine the pooling of data and the generalizability of the label. Thus, regulatory guidance has stressed the need for demonstrating that the treatment effect is robust and clinically meaningful in each relevant subgroup, or else justifying a homogeneous combined population.

Overall, both FDA and EMA require that RDoC-aligned trials adhere to the same core standards as any other trial: predefined hypotheses, valid and reliable measures, and evidence of benefit that can be clearly communicated in labelling. The novelty is not lost on regulators—for instance, FDA officials have acknowledged in commentary that entirely new frameworks may eventually warrant “new FDA guidance” once RDoC paradigms mature (Citrome et al., 2022). However, until such policies materialize, sponsors must work within existing regulatory frameworks, using the flexibilities available (e.g., enrichment strategies, ancillary endpoints, subgroup analyses) to pair the RDoC approach with the practical need for a definable indication and outcome.

In summary, our key recommendation to navigate regulatory challenges related to RDoC-based therapeutics is to engage early and often with regulators (e.g., seeking scientific advice meetings with EMA or Type C meetings with FDA). Proactive dialogue can help ensure that trial designs and endpoints are tuned to regulatory expectations for future approval by allowing regulators to provide non-binding feedback on critical questions (e.g., “Will the agency accept a certain symptom scale as a primary endpoint?” or “Is the proposed patient selection strategy feasible for an indication?“) before the sponsor is deep into a program (McCarthy and Gallo, 2024). It also enables the use of formal qualification pathways: both agencies encourage sponsors to pursue qualification for novel biomarkers or COAs that do not fit neatly into existing guidance. To this end, the FDA's Drug Development Tool qualification programs (for biomarkers, clinical outcome assessments, etc.) and its PFDD meetings can be leveraged to gain regulatory buy-in on the tools needed for RDoC-aligned trials (U.S. Food and Drug Administration, 2025; Drmić et al., 2024; U.S. Food and Drug Administration, 2020). Similarly, the EMA's Qualification of Novel Methodologies procedure offers a pathway for validating innovative endpoints or biomarkers for a defined context of use (European Medicine Agency, n.d.). A recent review of the program provided practical tips (like ensuring a well-defined context of use and robust validation data) for successfully achieving a qualification opinion (Drmić et al., 2024). By securing a regulatory “stamp” on a novel endpoint or patient-reported outcome measure, a sponsor greatly increases the chance that pivotal trials using those tools will be accepted as evidence.

## 7 Discussion

Across our analyses, a clear picture emerges regarding the application of the RDoC framework in clinical development. On the one hand, RDoC-aligned principles can inform more mechanistically coherent trial designs and increase signal detection. On the other hand, their implementation is hindered by a lack of terminological clarity, properly validated transdiagnostic constructs, FFP transdiagnostic COA, and regulatory frameworks that remain tied to categorical diagnoses. Together, these obstacles may explain why RDoC principles have been successfully applied in exploratory studies yet rarely sustained into pivotal development.

**Fig. 2:**
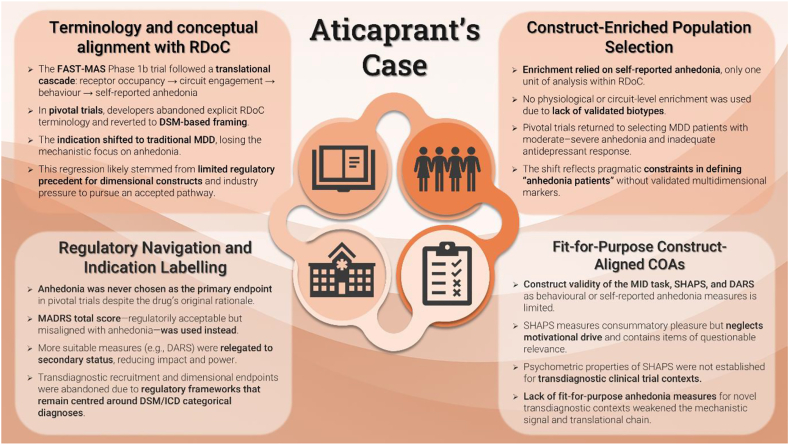
Aticaprant's development program: a critical view through the lens of the suggested key operational areas presenting significant barriers for the implementation of the Research Domain Criteria framework in clinical development.

Aticaprant's development programme illustrates both promise and pitfalls of applying RDoC principles in clinical development. Whereas the FAST-MAS trial showcased the framework's true potential to link mechanistic signals to clinical benefits, methodological and regulatory hurdles led to an abandonment of its principles in late-stage development, which may partly explain the disappointing Phase 3 results (Krystal et al., 2020; Johnson, 2025). Across the four operational areas presenting significant barriers for RDoC's operationalization, two main themes consistently emerged when analysing this case (Fig. 2; Supplementary Table S3). First, bridging anhedonia endpoints may not have been FFP in this novel (transdiagnostic) context of use to carry early mechanistic effects into late-phase, patient-centred benefits. Second, and more fundamentally, regulatory agencies currently do not recognise ‘anhedonia’ as an independent disease entity, therefore preventing construct-centric transdiagnostic treatment indications. This regulatory conservatism necessitated a reversion to conventional diagnostic boundaries and primary endpoints, thereby limiting the feasibility of sustaining RDoC-aligned strategies through to late-stage clinical evaluation and, eventually, regulatory approval.

Beyond aticaprant, other development programs illustrate similar translational and operational constraints when RDoC-relevant constructs are brought into early clinical development. For example, navacaprant (NMRA-140), another κ-opioid receptor antagonist with demonstrated antianhedonic properties in early-phase studies (Mathew et al., 2025), failed to separate from placebo in the KOASTAL-1 pivotal program where developers reverted to syndrome-level as primary endpoint. Instead, the construct-relevant readout for anhedonia (i.e., SHAPS) was included a secondary endpoint, yet demonstrating a significant treatment effect on anhedonic symptom reduction compared to the placebo (albeit in female participants only) (Neumora). Similarly, the ALTO-100 development programme illustrates both the ambition and fragility of biomarker-guided enrichment strategies: early signals supporting a ‘cognitive profile’ approach required prospective replication (Papakostas et al., 2019), and later biomarker-stratified trials did not meet the primary endpoint (Alto Neuroscience and Inc), reinforcing the need for rigorous pre-specification and cross-study validation of predictive signatures before they can credibly support a registrational strategy. Finally, the rTMS + ERP example in OCD emphasizes that mechanistic predictors (e.g., task-fMRI readouts) may be useful for early proof-of-mechanism and stratification but generally remain far from the evidentiary and standardization requirements needed for regulatory decision-making (Postma et al., 2025; Fitzsimmons et al., 2025).

We propose pragmatic solutions to overcome the operational barriers highlighted above. First, consensus-building activities should define classification criteria for RDoC-aligned trials to prevent terminological drifts and increase transparency and effective communication between stakeholders. Second, biomarker-based enrichment strategies, such as transdiagnostic biotypes, should be leveraged for construct-valid selection of clinical trial populations and to avoid pseudospecificity. Third, Clinical Outcome Assessments should be selected, developed, or modified according to current standards to qualify as FFP for novel transdiagnostic research settings, while considering patient-centric factors. And fourth, to meet current regulatory requirements for labelling, construct-based transdiagnostic indications should initially be anchored to a single recognised disease entity and subsequently expanded to other diseases after approval. Moreover, if novel RDoC-based methodologies are applied to clinical trials, developers should seek engagement with regulatory agencies early in the development process to increase the likelihood of obtaining acceptance.

Beyond what has been discussed in the present work, translation to true precision-psychiatry will require a “next-layer” refinement of RDoC constructs to define sharper, testable sub-construct definitions anchored to specific neural/cognitive mechanisms and linked to validated, transdiagnostic clinical outcomes. Without it, RDoC-aligned designs risk remaining descriptive instead of clinically meaningful and will continue to underdeliver on stratification and treatment-response prediction—the core promise of precision medicine. As such, construct-level granularity, supported by harmonized COAs and multimodal biomarkers/digital phenotypes, should be pursued in parallel with therapeutic development.

Reiterating our intention to distil pragmatic priorities arising from our discussions, there are additional issues related to the implementation of the RDoC framework that need to be mentioned. Above all, we are aware that implementing RDoC at scale faces significant feasibility barriers which may limit acceptance by Health Technology Assessment agencies (Morris et al., 2022; Akram and Giordano, 2017; Baldwin et al., 2022). For example, the need for specialised personnel and multi-modal instrument infrastructure may represent a significant cost barrier, especially to healthcare systems of low-income countries. Nevertheless, as recently demonstrated in adjacent areas (e.g., neurodegeneration imaging), methodological innovations with a clear value proposition will likely become routine once sufficient resources are allocated to establish standards, training, and shared platforms. Moreover, early interaction with Health Technology Assessment agencies will ensure that chosen endpoints capture outcomes meaningful to patients and payers and smoothen the translation from clinical evidence to value in health care (Kennedy-Martin et al., 2020; Kaplan and Hays, 2022).

As for precision psychiatry in general, implementing RDoC in routine clinical development would be a multi-year, likely even multi-decade, endeavour. To help coordinate investments and avoid duplication, there is the need to align current global precision psychiatry initiatives, shared core-outcome sets, and coordinated qualification efforts (FDA/EMA and beyond). Most notably, the Precision Psychiatry Roadmap advanced by the ECNP identifies harmonization of methodologies, biomarker validation, and adaptive trial architectures as priorities for advancing the field (Kas et al., 2025). Within the PPR, the RDoC framework constitutes the conceptual underpinnings that the proposed roadmap seeks to operationalize, with the goal of delivering tangible benefits to patients in real-world settings.

In sum, this work charts a path forward for embedding the RDoC framework into early-phase neuropsychiatric drug development—calling not only for greater methodological alignment and translational rigor, but for a paradigm shift in how we define, measure and intervene in brain disorders. While significant hurdles remain, this review underscores that those challenges also present fertile opportunities for innovation. The future of psychiatric drug development may well depend on embracing this precision-informed, domain-driven science; only then can promising interventions, like aticaprant, bring tangible and clinically meaningful benefits to the patients and the wider community.

## Contributions

All authors meet the International Committee of Medical Journal Editors (ICMJE) criteria for authorship, including substantial contributions to the work, critical revision of the content, final approval of the version to be published, and agreement to be accountable for all aspects of the manuscript. CRediT author contribution statement is as follows:

• Conceptualization: IM, RS, PH, JA, CJE, LP, FdC, SZD, JE
• Project administration: IM
• Writing – original draft: IM
• Writing – review & editing: IM, RS, PH, JA, CJE, LP, FdC, SZD, JE

Section-level contributions (within the roles above):

• RDoC in clinical development (conceptualization; review & editing): PH, RS, JA, CJE
• Regulatory considerations (conceptualization; review & editing): LP, FdC, JA
• Clinical outcome assessments (conceptualization; review & editing): CJE, SZD, JE
• Target population selection (conceptualization; review & editing): FdC, RS, JA

## Declaration of generative AI and AI-assisted technologies in the writing process

During the preparation of the final version of the manuscript the authors used GPT-3o and GPT-5 (OpenAI) for the sole purpose of improving fluency and readability of the text. After using this tool, the authors reviewed and possibly edited the content as needed. The authors take full responsibility for the content of this publication.

## Financial disclosures

This work was financially supported by the 10.13039/501100007871European College of Neuropsychopharmacology. IM, RS, PH, and JA have no conflicts of interest to declare. In the past 24 months, SZD has provided consultancy services to Lundbeck A/S, Sanofi, Jazz Pharmaceuticals and Merck Healthcare. SZD is also the developer of a novel instrument to measure CIAS in Schizophrenia (EPICOG-SCH). In the past 24 months, CJE has provided consultancy services to Cogstate (UK), Clinical Outcome Solutions (UK), Ardea Outcomes (CA), Vanqua Bio (USA), P1Vital (UK), Lighthouse Pharma (USA), and Boehringer Ingelheim (DE), and is a Cogstate shareholder. In the past 24 months, FdC received paid consulting/advisory engagements with relevant entities for the present manuscript contents: Atai Life Sciences (USA/Germany). LP is a part-time employee of the University of Miami (USA) and the University of Modena and Reggio Emilia (Italy). In the past 24 months, paid consulting/advisory engagements with relevant entities for the present manuscript contents are: AbbVie (USA), AiDvance (DE), Adapt (UK), BCG (CH), Boehringer Ingelheim International GmbH (DE), Compass Pathways (UK), EDRA-LSWR Publishing Company (IT), EnZeta (USA), Ferrer (ES), GH Research (IE), Immunogen (USA), Inpeco SA (CH), Johnson & Johnson (USA), Napo Pharma (USA and EU), NeuroCog Trials (USA), Novartis-Gene Therapies (CH), Sanofi-Aventis-Genzyme (FR and USA), NetraMark (CA), Otsuka (USA), Pfizer Global (USA), RAIN Scientific (USA), Relmada Therapeutics (USA), Takeda (USA), Vifor (CH), WCG-VeraSci (USA). Equity/Options: AiDvance; Adapt; EnZeta; Relmada; NetraMark; RAIN Scientific. JE is a full-time employee of Cronos Clinical Consulting Services, Inc. (an IQVIA business, USA); receives scale-related licensing fees for the Anhedonia Interview Rating Scale (AIRS, AIRS-SR). All authors declare no other relationships or activities that could appear to have influenced the submitted work. Author names and affiliations are listed in the title page of the manuscript.

## Footnotes

1 **Abbreviations:** Bipolar–Schizophrenia Network on Intermediate Phenotypes (B-SNIP); Clinical Outcome Assessment(s) (COAs); Concept of Interest (COI); Dimensional Anhedonia Rating Scale (DARS); Electroencephalogram (EEG); European College of Neuropsychopharmacology (ECNP); European Medicines Agency (EMA); Fit-For-Purpose (FFP); International Classification of Diseases (ICD); International Society for CNS Trials and Methodology (ISCTM); κ-Opioid Receptor (KOR); Major Depressive Disorder (MDD); Montgomery–Åsberg Depression Rating Scale (MADRS); Patient-Focussed Drug Development (PFDD); Research Domain Criteria (RDoC); Reward Task Optimisation Consortium (RTOC); Selective Serotonin Reuptake Inhibitor (SSRI); Snaith-Hamilton Pleasure Scale (SHAPS).

2 **The Research Domain Criteria (RDoC) framework**. Launched in 2010 by the U.S. National Institute of Mental Health (NIMH), RDoC is a translational research framework that reconceptualises mental disorders in terms of neurobiological and behavioural constructs rather than categorical diagnoses (Cuthbert and Insel, 2013; Insel et al., 2010; Morris et al., 2022). Constructs are organised into six functional domains (i.e., positive valence systems, negative valence systems, cognitive systems, sensorimotor systems, arousal/regulatory systems, systems for social processes) and are interrogated across multiple units of analysis—genes, molecules, cells, circuits, physiology, behaviour, and self-report—spanning the full health-to-illness continuum. Key principles are: (i) dimensionality—psychopathology is graded, not all-or-none; (ii) transdiagnostic scope—constructs cut across DSM/ICD categories; (iii) multi-level integration—evidence from brain to behaviour must converge; (iv) developmental and environmental context—construct expression changes across the lifespan and with experience. RDoC aims to foster mechanism-based hypotheses, enable biomarker discovery, and ultimately support precision interventions by linking specific neural systems to clinically meaningful outcomes.
