# Supplementary material for: Application of the research domain criteria in early-phase clinical development of transdiagnostic neurotherapeutics: A multidisciplinary perspective
Source: Neurosci Appl. 2026 Mar 7;5:106996. doi: 10.1016/j.nsa.2026.106996 (PMC13058995; doi:10.1016/j.nsa.2026.106996)
Supplement: Multimedia component 1 [file mmc1.docx]

# Supplementary Files

Supplementary Table S1. Expanded comparison of illustrative case studies for RDoC-aligned drug development efforts (mechanism/construct → operationalization → outcomes → translation/regulatory lessons).

| Intervention and Context of Use | Hypothesized mechanism | Early-phase alignment strategy | Late-stage constraints | Key outcome | Implication for RDoC-based development | Key references |
| --- | --- | --- | --- | --- | --- | --- |
| Aticaprant  (JNJ-67953964; kappa-opioid receptor antagonist)  Adjunctive treatment in major depressive disorder (MDD); focus on anhedonia | Positive Valence Systems (reward responsiveness / reward learning), with stress–reward interactions.  Hypothesis: blocking dynorphin–kappa signaling reduces stress-induced dysphoria and normalizes reward circuitry. | Proof-of-mechanism FAST-MAS program linked drug exposure to changes in reward-related neural activity and anhedonia measures.  Phase 2 trial used placebo lead-in non-responder enrichment and stratified by baseline hedonic capacity (Snaith–Hamilton Pleasure Scale). | VENTURA Phase 3 adjunctive program in MDD with moderate–severe anhedonia.  Primary endpoint anchored to global depressive symptom severity (Montgomery–Åsberg Depression Rating Scale total score), with conventional inclusion/exclusion constraints typical of registration studies. | Phase 2: statistically significant (modest) improvement vs placebo on depressive symptom total score; larger effect in higher-anhedonia subgroup.  Phase 3: program discontinued due to insufficient efficacy in target population (full analyses pending public presentation). | Even when a construct (anhedonia) is prospectively targeted, pivotal programs may still rely on broad syndrome-level endpoints; modest Phase 2 effects can evaporate under phase 3 constraints and placebo response.  Pre-specify a bridging strategy that ties mechanistic/construct readouts to registration-relevant endpoints and functional outcomes, and pursue early regulatory alignment on what constitutes clinically meaningful improvement for the construct. | [21,26,27] |
| Navacaprant  (NMRA-140; kappa-opioid receptor antagonist)  Monotherapy in MDD; focus on anhedonia | Positive Valence Systems (hedonic capacity/reward responsiveness).  Hypothesis: kappa antagonism modulates reward pathways and dopamine signaling relevant to anhedonia. | Phase 2a explicitly included hedonic capacity as a secondary endpoint (Snaith–Hamilton Pleasure Scale) alongside syndrome-level severity.  Program rationale emphasized differentiated impact on anhedonia beyond overall mood symptoms. | KOASTAL pivotal program: primary endpoint change in Montgomery–Åsberg Depression Rating Scale total score at Week 6; key secondary SHAPS.  Pivotal program designed for NDA-style evidence generation; high placebo response risk typical in MDD. | Phase 2a: did not separate from placebo on syndrome-level primary endpoint at Week 8; exploratory signals varied across measures.  KOASTAL-1 Phase 3: no statistically significant difference vs placebo on MADRS or SHAPS at Week 6 (sex-differential signal observed; further analyses planned). | A construct-relevant secondary endpoint (anhedonia) does not protect against failure if the pivotal trial is powered and interpreted primarily through syndrome-level outcomes.  Highlights the need for (i) robust construct-sensitive effect sizes, (ii) prospectively defined enrichment/biotyping to reduce heterogeneity, and (iii) early agreement with regulators on hierarchy/interpretation of construct endpoints. | [29,30] |
| Brexanolone  (allopregnanolone; GABAA receptor positive allosteric modulator)  Intravenous treatment for postpartum depression (PPD) | Arousal/Regulatory Systems and Negative Valence Systems.  Hypothesis: neurosteroid modulation of GABAA signaling restores stress–arousal regulation linked to postpartum mood symptoms. | Mechanistically anchored to a neurosteroid/hormonal transition model; clinical program used a time-limited infusion with rapid symptom change.  Did not require an RDoC biomarker but represents a biologically specific hypothesis that translated to clear clinical readouts. | Pivotal Phase 3 trials used standard clinician-rated depressive severity outcome (Hamilton Depression Rating Scale) in DSM-defined PPD.  Regulatory evaluation centered on conventional symptom outcomes and safety monitoring (e.g., sedation). | Phase 3 trials showed rapid and sustained improvement vs placebo on depressive symptom scales.  FDA approval was granted; product later discontinued from marketing and approval was withdrawn upon request (commercial—not efficacy—context). | Counter-example where a biologically grounded hypothesis succeeded because the program maintained clear clinical endpoints acceptable to regulators.  Also illustrates that translation success is not only scientific/regulatory—implementation constraints (administration burden, monitoring) can shape real-world impact. | [31,32] |
| ALTO-100  (NSI-189 phosphate; neurogenic small molecule)  MDD; biomarker-guided ‘cognitive profile’ strategy (company program) | Cognitive Systems (learning/memory, cognitive control) and neuroplasticity.  Hypothesis: enhancing hippocampal neurogenesis / neuroplasticity improves cognitive-affective dysfunction dimensions. | Earlier development (Neuralstem) used conventional MDD scales plus patient-rated dysfunction (cognition/physical function) and exploratory biomarkers.  Alto strategy layered biomarker-defined subgrouping (EEG/cognitive/wearable profiles) to identify patients more likely to respond. | Phase 2b program tested efficacy in a predefined biomarker-positive subgroup with standard depressive symptom endpoint (MADRS).  Program illustrates ‘biotyping’ used to preserve mechanistic intent while still using regulatory-recognized symptom outcomes. | Neuralstem Phase 2 (SPCD): primary endpoint on MADRS not met; some secondary self-rated measures improved.  Alto: open-label signal in biomarker-positive group reported; subsequent randomized Phase 2b did not meet the primary endpoint in the biomarker-positive subgroup. | Biomarker enrichment can be necessary but not sufficient; subgroup definitions must be prospectively robust, reproducible, and operationalizable at scale.  If pivotal endpoints remain syndrome-level (MADRS), the biomarker must demonstrate a strong, consistent interaction with treatment effect—otherwise the ‘RDoC layer’ may not translate. | [33–35] |
| rTMS  (High-frequency rTMS delivered immediately before exposure and response prevention (ERP) therapy for OCD) | Cognitive Systems (cognitive control; inhibitory control) and Negative Valence Systems (threat-related responding).  Hypothesis: rTMS-induced plasticity boosts cognitive control circuitry, increasing learning from ERP and symptom reduction. | Mechanistic design: synchronizing neuromodulation with a learning-based intervention (ERP).  Neural predictors derived from cognitive-control task signals/connectivity, aiming to identify who benefits most. | Clinical outcomes still anchored to symptom change (Y-BOCS) and standard response definitions.  If scaled, implementation requires feasible biomarker acquisition and clinically deployable stimulation/therapy workflows. | Small mechanistic trial suggests that baseline neural markers of cognitive control predict symptom improvement when rTMS precedes ERP; effect requires replication.  Provides an example of ‘construct-to-intervention timing’ rather than diagnosis-only matching. | Illustrates how construct-linked mechanistic hypotheses can be embedded in study design (timing + biomarkers) and connected to clinically meaningful outcomes.  For RDoC translation, mechanistic predictors should be explained in terms of actionable circuit functions (e.g., cognitive control capacity) and validated prospectively for scalability. | [36,37] |

Abbreviations: MADRS = Montgomery–Åsberg Depression Rating Scale; HAMD/HAM-D = Hamilton Depression Rating Scale; SHAPS = Snaith–Hamilton Pleasure Scale; TRD = treatment-resistant depression; PPD = postpartum depression; rTMS = repetitive transcranial magnetic stimulation; ERP = exposure and response prevention; Y-BOCS = Yale–Brown Obsessive Compulsive Scale; RDoC = Research Domain Criteria.

**Supplementary Table S2.** Potential validation hotspots for RDoC / transdiagnostic Clinical Outcome Assessment (COA) tools, as discussed during semi-structured online discussion meetings.

| VALIDATION HOTSPOT | RATIONALE |
| --- | --- |
| Conceptual/Content validity | RDoC constructs (e.g., “loss”, “cognitive control”) transcend DSM labels; a single COI must resonate with patients who differ in diagnosis, stage and culture. Establishing that every item in an OMI is *relevant, comprehensive and comprehensible* for *all* sub-populations is considerably harder than in a single-disorder COS. |
| Fit-for-Purpose (FFP) versus clinical meaningfulness | Early-phase RDoC studies often need biomarker-like, high-sensitivity endpoints; regulators may later require proof that the same measure maps onto “how patients feel or function” in heterogeneous samples. Balancing these twin pathways is tricky. |
| Measurement invariance & psychometric robustness | Dimensional scores must show *reliability, construct and known-groups validity* across diagnoses, languages and digital formats. Proving invariance often demands very large, stratified samples—difficult in Phase I–II. |
| Minimal Clinically Important Difference & responder definition | Baseline functioning and variance differ greatly between, say, PTSD and MDD patients; a single Minimally Important Difference may misclassify benefit. Regulators still expect a credible MCID per COU. |
| Sample-size & enrichment logistics | Demonstrating psychometrics in every transdiagnostic subpopulation inflates N; yet early trials are resource constrained. |
| Cross-cultural & linguistic adaptation | A transdiagnostic COS almost always becomes multinational. Ensuring semantic and conceptual equivalence across cultures, literacy levels and clinical contexts is labour-intensive. |
| Regulatory pathway ambiguity | Neither FDA nor EMA have settled precedents for dimensional endpoints spanning multiple ICD codes; sponsors might face uncertainty on evidentiary standards. |
| Digital Health Technology Tools (DHTTs) | Passive capture of RDoC constructs (e.g., reward-related phone use) is attractive but typically sits at low validation level; linking the sensor signal to a patient-centred outcome and ensuring data privacy both need rigorous proof. |
| Innovation versus standardisation tension | Once a COS is “locked”, innovation can stall—yet RDoC science evolves rapidly. |

**Supplementary Table S3.** Detailed analysis of aticaprant’s development programme across the 4 operational areas identified as key for the operationalisation of RDoC in clinical development.

| Operational area | Rationale |
| --- | --- |
| TERMINOLOGy and definitions | Aticaprant’s early development aligned closely with RDoC's dimensional principles. In particular, the Phase 1b FAST-MAS trial targeted the Reward Responsiveness subconstruct of the Positive Valence domain, tracing a translational cascade covering various levels of analysis from receptor occupancy to circuit engagement, behavioural changes, and self-reported reductions in anhedonia. However, in pivotal trials, the terminological and conceptual link to RDoC was abandoned. Developers defaulted to a single DSM-defined MDD population; and a traditional regulatory accepted and gold standard primary depression endpoint (measured with the Montgomery–Åsberg Depression Rating Scale; MADRS), with anhedonia endpoints being secondary. This shift—likely driven by the lack of regulatory precedent and commercial considerations—significantly diluted the initial alignment with the RDoC framework. |
| Target Population | Consistent with RDoC's transdiagnostic principle, the FAST-MAS trial enriched participants based on anhedonia severity across different mood and anxiety disorders, using self-reported anhedonia as inclusion criteria. The multidimensional nature of RDoC constructs was not fully leveraged for population definition, since the enrichment strategy did not integrate markers across units of analysis, such as physiological or circuit-level indicators. At the time, the field lacked validated methods for stratifying patients by reward-system dysfunction, and unlike psychosis (e.g., B-SNIP biotypes), no equivalent biotyping efforts were available for depression/anhedonia. In the pivotal trials, developers reverted to select sample with MDD with Moderate-to-Severe Anhedonia due to inadequate response to the current antidepressant, effectively collapsing the construct-based enrichment strategy. This regression may be the result of pragmatic considerations: without a validated construct-based enrichment strategy, “anhedonia patients” could not be reliably or credibly defined at scale in clinical trials. |
| Clinical Outcome Assessments | In our view, the COA strategy was a central weakness in aticaprant’s development. During our discussions, concerns repeatedly surfaced regarding both the choice and adequacy of anhedonia measurement instruments used in the clinical trials. For example, the Monetary Incentive Delay task (MID) used in the FAST-MAS trial as behavioural outcome measures for anhedonia was criticized for uncertain validity in capturing hedonic capacity (Bill Potter, personal communication). Similarly, the Snaith-Hamilton Pleasure Scale (SHAPS; Snaith et al., 1995) that was used to measure self-reported hedonic tone along the trial, was repeatedly flagged for psychometric limitations. Namely, the SHAPS is known for its ability to measures consummatory pleasure but neglects motivational drive. Moreover, several items on the scale have been criticised due to dubious clinical or personal relevance (e.g., “I would find pleasure in the scent of flowers or the smell of a fresh sea breeze, or freshly baked bread”). Despite being validated starting from surveys to general population, the psychometric properties of the SHAPS were not confirmed in this novel context of use; a limitation that was recognized by the authors (Snaith et al., 1995). Therefore, performance of the SHAPS among physically ill people should be established using other instruments for concurrent validity, recognizing that the ability to experience pleasure is certainly an important aspect for quality of life.  Despite aticaprant being advanced as a treatment for anhedonia initially, the construct was never selected as the primary endpoint in pivotal trials; a decision which was likely driven by regulatory-acceptable labelling strategies. In addition, a secondary measure of interest, motivation, effort and pleasure was added, the Dimensional Anhedonia Rating Scale (DARS; Rizvi et al., 2015), potentially addressing some of the aforementioned concerns regarding the SHAPS, but placing this one lower in the hierarchy, therefore lowering? chances to find significant results. This decision may reflect perceptions that available instruments were too narrow, insufficiently meaningful to patients, overly reliant upon self-report versus clinician-reported outcomes standard in psychiatry trials or carried a low probability of regulatory success. Instead, developers defaulted to the MADRS total score, a regulatory accepted but construct-misaligned endpoint when targeting the motivational component of mood disorders, i.e. MADRS only offers one single item dedicated to anhedonia, item 8 as “inability to feel” in the sense of capacity to react to others, which includes lack of capacity of feeling anger, grief and pleasure. To summarize, without a FFP regulatory-accepted COAs for anhedonia, construct-driven development of aticaprant for anhedonia failed. These considerations highlight the relevance of setting a feasible COA strategy early in the drug development process, when Phase 1 trials are being planned and in agreement with regulators in order to collect the appropriate data for qualification of the endpoints included in the study design. |
| Regulatory Aspects | Regulatory hurdles may have decisively shaped aticaprant’s clinical development trajectory and prevented alignment with RDoC’s principles in late-phase trials. In fact, attempting a purely construct-based transdiagnostic label, like anhedonia, would still be deemed too risky given the absence of regulatory precedent. Reflecting this conservatism, anhedonia measures were used as secondary/exploratory endpoints in pivotal trials, while conventional depression scales (i.e., MADRS) were used as primary. In fact, while early anhedonia readouts (e.g., MID, SHAPS) were useful for de-risking in Phase 1/2, they lacked formal qualification and thus could not support approval. Similarly, transdiagnostic recruitment across DSM conditions was abandoned in favour of a single MDD subpopulation. These decisions support the view that implementing RDoC in late-stage clinical development requires careful considerations of current regulatory frameworks. |
